# Investigating the incremental value of urine sediment reporting in emergency medicine with a Sysmex UN urinalysis system

**DOI:** 10.1515/almed-2024-0035

**Published:** 2024-07-12

**Authors:** Marco Tosi, Davide Negrini, Giovanni Celegon, Martina Montagnana, Giuseppe Lippi

**Affiliations:** Section of Clinical Biochemistry and School of Medicine, 9286 University of Verona, Verona, Italy

**Keywords:** urinalysis, urinary sediment analysis, emergency medicine, clinical laboratory techniques

## Abstract

**Objectives:**

Urinalysis is widely used and is also frequently requested in emergency situations for screening hypovolemia, urinary tract infections, diabetes, ketoacidosis and hematuria. Our aim was to evaluate the impact of reporting urinary sediment in emergency department specimens with the Sysmex UN system.

**Methods:**

We evaluated urinalyses requested by the emergency department over a three-month period and examined red blood cell count interference, compared leukocyte esterase dipsticks to cytofluorimetric leukocyte count and nitrites to cytofluorimetric bacterial count. We then examined digital microscopy images to identify additional elements of interest or pathology.

**Results:**

We collected 532 cases, 354 with only chemical and cytofluorimetric analysis and 178 with digital microscopy. Automated erythrocyte counting showed a 7 % error rate, mainly false-positive results. Leukocyte esterase had a sensitivity of 88.22 % and specificity of 88.84 % at the lower limit, while nitrites had a sensitivity of 41.06 % and a specificity of 99.38 %. Pathological elements were detected in 126 samples by digital microscopy: 70 had casts, 36 crystals and seven cells with high pathological value.

**Conclusions:**

Evaluation of urine sediments by trained specialists can provide potentially important information even in emergency situations, whereby the pre-analytical phase must always be taken into account.

## Introduction

Urinalysis is a widely used test because it can provide important clinical information, the sample collection is relatively simple, the test can be performed safely and accurately in almost any laboratory, and it has a favorable cost-benefit profile. Together with creatinine determination and calculation of the estimated glomerular filtration rate (eGFR), urinalysis is the first approach to the diagnosis of kidney disease and urinary tract injury or dysfunction [1, 2].

Urinalysis usually includes some or all of the following: physical examination (color and appearance), determination of urine concentration (relative density or osmolality), chemical measurements (proteins, glucose, ketones, hemoglobin, leukocyte esterase, nitrite, pH and other tests), examination of urine sediment (using automated analyzers to count corpuscular components and/or microscopic analysis for more detailed analysis of various elements detected) [3, 4].

In clinical practice, urinalysis is usually performed in case of suspected urinary tract infection, screening or follow-up of kidney disease, non-infectious urinary tract diseases (primary or secondary to systemic diseases, including rheumatic diseases, hypertension, other diseases of pregnancy or drug side effects), and in case of recurrent kidney stones formation [3, 5].

Urinalysis is also frequently requested in the emergency setting, mainly as a screening tool, for evaluation of states of dehydration and hypovolemia (e.g., assessing urine concentration) [6], in case of suspected urinary tract infections (owing to the high predictive value of leukocyte esterase and nitrite positivity) [7], in case of diabetes and diabetic ketoacidosis (assessing concentration of glucose and ketones), and for investigating blood loss from the urinary tract (i.e., hematuria). Several other clinical conditions can be investigated with urine chemistry tests, thus providing useful information for an accurate and relatively fast differential diagnosis [8].

In particular, all urinalyses should be requested on the basis of a specific clinical suspicion, as inappropriate requests may complicate test evaluation [9]. While physical and chemical tests are always performed in most clinical laboratories, evaluation of urine sediment in emergency situations is usually only performed when specifically requested. Potentially useful information that cannot be obtained from urine dipstick results may hence be lost.

The aim of this study was not to evaluate Sysmex UN instrumentation performances (already well documented in literature) but to evaluate the potential impact (in terms of the number of samples that need to be checked, modified or commented by an operator) of including also urinary sediment reporting in samples received from the local emergency department (for which we are currently reporting only the dip-stick results), as well as the concordance of some useful parameters between dipstick and cytofluorimetric test results (on which many cross-check rules are based), using a Sysmex UN urinalysis system based on a chemical analyzer, a cytofluorimetric platform (to screen the urinary sediments and reduce the number of samples needing microscopic evaluation), and an automated microscope.

## Materials and methods

### Data collection

In this retrospective observational study, we evaluated all the urinalysis requests placed by the emergency department of University Hospital of Verona during a 3-months period (from February to April 2023). For each request, the analysis was performed using a Sysmex UN-series modular system (Sysmex Corporation, Kobe, Japan), consisting of Sysmex UC-3500 (automated chemistry analyzer), using MediTape UC-11A test strips, Sysmex UF-4000 (cytofluorimetric method) and Sysmex UD-10 (digital microscopy unit). Urinary samples come from the emergency department to the laboratory by pneumatic tube transport and then processed within 30 min.

All analyses were performed with UC-3500 and UF-4000 analyzers. All samples with specific parameters or their combinations were also examined using UD-10 digital microscopy to obtain 40 bright-field images of urinary sediment for morphological evaluation. Two urinalysis-trained laboratory professionals reviewed the data and images of all samples and agreed on interpretation of test results.

The data collected for this study encompassed all results obtained using the instrumentation, extracted with the DMS-ANUR software (Dasit Group, Milan, Italy), with the addition of any corrections added before reporting, as well as comments added after morphological assessment of urine sediment, when necessary.

The rules adopted by our Laboratory on the Sysmex UN middleware (DMS-ANUR) for starting automated digital microscopy are reported in Supplementary Table S.1; the list of parameters collected in the study, reference range and current reporting status are represented in Supplementary Table S.2.

The urine sediment analysis report includes the number of erythrocytes (when >15/µL), leukocytes (when >20/µL), bacteria (when >1,000/µL), and squamous epithelial cells (when >20/µL), as provided by the analyzer. The number of elements for all other categories is not included in the final report and is only useful for flag raising and digital image evaluation. Thus, these other elements of potential clinical interest have been reported as descriptive comments with semi-quantitative evaluations (rare, some, numerous, “carpet”).

### Interference in the erythrocyte count in the SF_RBC/X’TAL channel

The scattergram of erythrocytes (RBCs) is vulnerable to interference [10, 11], appearing as an abnormal distribution of erythrocytes, showing scatter or ‘hook’ graphs. The SF_RBC/X’TAL scatter constructs a graph between depolarized side scattered light intensity and forward scattered light intensity, for distinguishing red blood cells (located at a low intensity zone of depolarized side scattered light) from crystals (which depolarize the light), but sometimes it can be interfered by low-depolarizing crystals or other particles. The number of samples in which the number of erythrocytes/µL provided by the instrument was changed due to interference in the count was then examined.

### Comparison of leukocyte esterase dipstick with cytofluorimetric leukocyte counts

Given the prevalence of false negatives, the added value of cytofluorimetric leukocyte counts over test strip staining in the evaluation of leukocyte esterase was evaluated [12]. Sensitivity and specificity were assessed at two levels: the first at the cut-off of positivity (1+ or higher) for the dipstick and 20 elements/µL for the leukocyte count in cytofluorimetry; the second at higher values with dipstick positivity at least classified as 2+ and 75 elements/µL (manufacturer’s parameter for 2+ test strip positivity) for leukocytes in cytofluorimetry.

### Comparison of dipstick nitrites with cytofluorimetric bacterial counts

The incremental value of the cytofluorimetric bacterial count was evaluated in comparison to the nitrite test strip staining, taking into account the frequent presence of false negatives because, as previously reported, not all bacteria produce nitrites. Sensitivity and specificity were thus assessed at the presence cut-off level, i.e. positive for dipstick, 1,000/µL for bacteria in cytofluorimetry (threshold defined in the Laboratory for ‘rare’).

### Other elements of interest or pathology (digital microscopy)

Samples underwent digital microscopy to identify additional elements of interest or pathology to the automatic quantification categories (erythrocytes, leukocytes, bacteria, squamous epithelial cells).

## Results

### Data collection

During the 3-month observation period, 532 cases from 265 male and 267 female emergency department patients were reviewed, with a mean age of 64.02 (standard deviation: 22.54) and median age of 69 (25th–75th percentile: 49–83) years. A total number of 354 out of the 532 samples were analyzed using only the UC-3500 and UF-4000, while the remaining 178 were also analyzed using digital microscopy with the UD-10. In 126 of the 178 test requests evaluated with UD-10 images, the presence of figured elements other than erythrocytes, leukocytes, bacteria, and squamous cells was deemed to be accompanied by a specific comment.

### Interference in the erythrocyte count in the SF_RBC/X’TAL channel

There were 37 errors (6.95 % of the total) in automated erythrocyte counting: 26 false positives (4.89 % of all requests; 70.27 % of errors), due to interference, in which erythrocytes were detected by the instrumentation despite their absence; 11 overestimation errors (2.06 % of all requests; 29.73 % of errors) were caused by abnormal erythrocyte distribution on the scattergram.

### Comparison of leukocyte esterase dipstick with cytofluorimetric leukocyte counts

At the presence cut-off (1+ for leukocyte esterase and >20/µL for the cytofluorometric count), the sensitivity was 88.22 % and the specificity was 88.84 %. The sensitivity was 91.18 % and the specificity was 94.17 % at a higher cut-off of leukocyte presence (2+ for leukocyte esterase and 75/µL at cytofluorimetric count).

### Comparison of dipstick nitrites with cytofluorimetric bacterial counts

The sensitivity at the presence cut-off was 41.06 %, with 99.38 % specificity.

### Other elements of interest or pathology (digital microscopy)

Elements of interest or pathology were detected in 126 out of the 178 samples examined (71 %) using digital microscopy: 70 samples had casts (43 hyaline casts and 27 had more pathological casts, such as hyaline-granular and granular, with the presence of waxy casts detected in one case).

As concerns the presence of non-squamous epithelial cells (39 samples), 32 samples had low pathological value cells (superficial transitional cells), while seven had higher pathological value cells (deep transitional, upper tract, renal tubular). Yeasts were found in 11 different samples. Crystals were found in 36 samples, 20 of which were calcium oxalate, 11 of which were triple magnesium ammonium phosphate, and five of which were uric acid. The presence of sperm contamination was identified in a single sample.

## Discussion

Although urinalysis is primarily a screening test, it outlines a profile of several parameters that provide a wealth of information on the possibility of kidney and/or urinary tract diseases, as well as systemic pathologies [13]. The current levels of automation and good analytical performance have shifted the focus to the need to optimize the pre-analytical phase in order to obtain accurate results. Optimal sample collection conditions are not always achieved, particularly in an emergency setting [14], predisposing to false positivity or negativity of the various tests performed.

The evaluation of interference in the erythrocyte count performed with the cytofluorimetric system allowed us to highlight the need for a revision performed by an experienced operator before the result is released in all cases where the value reported by the dipstick and the automated count are inconsistent. In the case histories we have examined, approximately 7 % of the samples had an issue with the automated erythrocyte count being overestimated, resulting in a false positive result in the majority of cases.

A comparison of leukocyte esterase assessment by dry chemistry (dipstick) and cytofluorimetric analysis of leukocyturia was also performed, confirming the good accuracy of the reactive pad, both at the positivity cut-off (sensitivity of 88 % and specificity of 89 %) and at higher cut-offs (where both sensitivity and specificity were above 90 %).

On the other hand, the dipstick assessment of nitrites (a surrogate for the presence of bacteria in urine) performed poorly, with a sensitivity of only 41 % at the positivity cut-off. This low sensitivity could be explained by the fact that not all bacteria that cause urinary infections are nitrate-reducers. Because the specificity was high (99 %), a positive result on the nitrite reactive pad indicates the presence of bacteria in the test sample with a high degree of certainty.

The digital microscopy analysis of sediment provided interesting evidence because figured elements with clinical significance could be detected in the majority of samples (71 %), thus necessitating a descriptive comment in the final report. Because there are no reactive pads that can detect these elements, the use of the dipstick alone is not a suitable approach, potentially masking a pathological condition. It would be useful, for example, to alert the clinicians to the presence of clearly pathological crystals, such as magnesium ammonium triple-phosphate crystals, which are commonly found in cases of genital-urinary tract infections, or to identify possible crystalluria in patients with suspected kidney stones disease. Further investigations may be required in these cases.

The assessment and reporting of morphological components in urine sediment, even in an emergency situation, is, therefore, a useful addition that not only improves the overall performance of the urinalysis test but also provides the clinician with additional, potentially important information. Of course, the request must be motivated and the pre-analytical phase must be rigorous in order to collect samples that can provide valuable clinical information.

## Supplementary Material

Supplementary Material
