# Supplementary material for: Investigating the incremental value of urine sediment reporting in emergency medicine with a Sysmex UN urinalysis system
Source: Adv Lab Med. 2024 Jul 12;5(4):377–80. doi: 10.1515/almed-2024-0035 (PMC11661545; doi:10.1515/almed-2024-0035)
Supplement: Supplementary file 1 — Supplementary Material [file j_almed-2024-0035_suppl_001.docx]

**Supplementary material**

**Table 1:** Rules adopted by our Laboratory on the Sysmex UN middleware (DMS-ANUR) for starting automated digital microscopy.

| **Rule** |
| --- |
| Search for magnesium ammonium triplo-phosphate crystals: pH > 8 + Bacteria > 1000 + Casts > 1 or Crystals > 10 |
| Spermatozoa ≥50 + Protein ≥15 |
| Crystals ≥30 |
| Crystals ≥15 + pH 5.0 or 5.5 or 6.0 |
| Atypical cells ≥5 |
| Non-Squamous Epithelial Cells ≥10 |
| TranEC or RTEC ≥8 cells |
| Hyaline casts ≥3 |
| Hyaline casts ≥2 + Protein present |
| Pathological casts ≥1.5 |
| Pathological casts ≥1.0 + Protein present |
| Yeasts ≥30 |
| Glucose 500 or 1000 + Yeasts 10 - 30 |
| Appearance Cloudy + Negative sediment |
| Esterase negative + Leucocytes ≥150 |
| Leucocytes < 20 + Esterase 2+ or 3+ |

**Table 2**: List of parameters collected in the study, reference range and current reporting status.

| **Parameter** | **Units of measurement** | **Reference range** | **Reporting** |
| --- | --- | --- | --- |
| **Chemical (chemical-physical) examination** | | | |
| Haemoglobin | mg/dL | <0,03 | Reported in routine and emergency |
| Leucocyte esterase |  | Absent | Reported in urgency |
| Nitrites |  | Absent | Reported in routine and emergency |
| Glucose | mg/dL | <10 | Reported in routine and emergency |
| Ketones | mg/dL | <5 | Reported in routine and emergency |
| Bilirubin | mg/dL | <0,2 | Not reported |
| Urobilinogen | mg/dL | <0,5 | Not reported |
| Creatinine | mg/dL | 20-300 | Not reported |
| Protein | mg/dL | <15 | Reported in routine and emergency |
| Protein/Creatinin Ratio | mg/gCr | <150 | Reported in routine and emergency |
| Albumin | mg/L | <20 | Not reported |
| Albumin/Creatinine Ratio | mg/gCr | <30 | Reported in routine and emergency |
| pH |  | 5,5-7,5 | Reported in routine and emergency |
| Relative Density (Specific Weight) |  | 1,005-1,030 | Referred in routine and emergency |
| Colour |  | Yellow | Reported in routine and emergency (only if abnormal) |
| Appearance |  | Limpid | Not reported |
| **Morphological examination (sediment)** | | | |
| Erythrocytes | n°/ µL | 0-15 | Reported in routine |
| Leukocytes | n°/ µL | 0-20 | Reported in routine |
| Bacteria | n°/ µL | Absent | Reported in routine |
| Squamous Cells | n°/ µL | 0-20 | Reported in routine |
| Non-Squamous Cells | n°/ µL | 0-8 | Not reported |
| Atypical Cells | n°/ µL | 0-5 | Not reported |
| Hyaline casts | n°/ µL | 0-2 | Not reported |
| Pathological casts | n°/ µL | 0-1 | Not reported |
| Crystals | n°/ µL | 0-15 | Not reported |
| Yeasts | n°/ µL | 0-30 | Not reported |
| Spermatozoa | n°/ µL | 0-50 | Not reported |
| Mucus | n°/ µL | 0-40 | Not reported |
| Conductivity | mS/cm | 3-38 | Reported in routine and emergency |
